# Surface Functionalization of Titanium-Based Implants with a Nanohydroxyapatite Layer and Its Impact on Osteoblasts: A Systematic Review

**DOI:** 10.3390/jfb15020045

**Published:** 2024-02-16

**Authors:** Karolina Homa, Wojciech Zakrzewski, Wojciech Dobrzyński, Paweł J. Piszko, Aleksandra Piszko, Jacek Matys, Rafal J. Wiglusz, Maciej Dobrzyński

**Affiliations:** 1 Niepubliczny Zakład Opieki Zdrowotnej Medident, Żeromskiego 2A, 43-230 Goczalkowice-Zdroj, Poland; kakahoma@poczta.onet.pl; 2 Department of Pediatric Dentistry and Preclinical Dentistry, Wroclaw Medical University, Krakowska 26, 50-425 Wroclaw, Poland; pawel.piszko@pwr.edu.pl (P.J.P.); aleksandra.piszko@student.umed.wroc.pl (A.P.); maciej.dobrzynski@umw.edu.pl (M.D.); 3 Pre-clinical Research Centre, Wroclaw Medical University, Bujwida 44, 50-368 Wroclaw, Poland; wojciech.zakrzewski1992@gmail.com; 4 Department of Dentofacial Orthopedics and Orthodontics, Division of Facial Abnormalities, Wroclaw Medical University, Krakowska 26, 50-425 Wroclaw, Poland; wojt.dobrzynski@wp.pl; 5 Department of Polymer Engineering and Technology, Faculty of Chemistry, Wroclaw University of Science and Technology (WUST), Wyb. Wyspianskiego 27, 50-370 Wroclaw, Poland; 6 Oral Surgery Department, Wroclaw Medical University, Krakowska 26, 50-425 Wroclaw, Poland; 7 Department of Organic Chemistry, Bioorganic Chemistry and Biotechnology, Faculty of Chemistry, Silesian University of Technology, Krzywoustego 4, 44-100 Gliwice, Poland; 8 Institute of Low Temperature and Structure Research, PAS, Okolna 2, 50-422 Wroclaw, Poland

**Keywords:** nanohydroxyapatite, surface coating, osseointegration

## Abstract

This study aims to evaluate the influence of a nanohydroxyapatite layer applied to the surface of titanium or titanium alloy implants on the intricate process of osseointegration and its effect on osteoblast cell lines, compared to uncoated implants. Additionally, the investigation scrutinizes various modifications of the coating and their consequential effects on bone and cell line biocompatibility. On the specific date of November 2023, an exhaustive electronic search was conducted in esteemed databases such as PubMed, Web of Science, and Scopus, utilizing the meticulously chosen keywords ((titanium) AND ((osteoblasts) and hydroxyapatite)). Methodologically, the systematic review meticulously adhered to the PRISMA protocol. Initially, a total of 1739 studies underwent scrutiny, with the elimination of 741 duplicate records. A further 972 articles were excluded on account of their incongruence with the predefined subjects. The ultimate compilation embraced 26 studies, with a predominant focus on the effects of nanohydroxyapatite coating in isolation. However, a subset of nine papers delved into the nuanced realm of its modifiers, encompassing materials such as chitosan, collagen, silver particles, or gelatine. Across many of the selected studies, the application of nanohydroxyapatite coating exhibited a proclivity to enhance the osseointegration process. The modifications thereof showcased a positive influence on cell lines, manifesting in increased cellular spread or the attenuation of bacterial activity. In clinical applications, this augmentation potentially translates into heightened implant stability, thereby amplifying the overall procedural success rate. This, in turn, renders nanohydroxyapatite-coated implants a viable and potentially advantageous option in clinical scenarios where non-modified implants may not suffice.

## 1. Introduction

Titanium and its alloys play a crucial role in medical and dental applications, particularly in the creation of orthopaedic and dental implants [1]. Recognized for its outstanding biocompatibility, which promotes strong cell attachment, high mechanical strength, and corrosion resistance, titanium stands out as a material with exceptional properties [1,2,3], hence its widespread adoption in contemporary dentistry. Dental titanium has emerged as the preferred material for applications such as dental implants, consistently demonstrating high success rates over the years. In recent decades, there has been a surge in the development of various methods to modify the surface of titanium dental implants, aiming to enhance both its physical and chemical characteristics. This pursuit is driven by the desire to improve patient comfort, reduce healing periods, and achieve more predictable treatment outcomes [4]. Despite titanium’s inherent excellence, surface modifications offer the potential to further elevate treatment results. Techniques such as roughening, air abrasion, acid etching, titanium plasma spray (TPS), micro-arc oxidation (MAO), and steam–hydrothermal treatment (SHT) are among the methods employed for surface functionalization. Titanium plasma spray (TPS) stands out as a commonly used approach [1]. Notably, additive manufacturing (AM) holds significant promise, allowing for the replication of natural cancellous bone structure [2]. Nevertheless, it is crucial to note that while dental implants serve as an excellent solution for replacing missing teeth, they do not offer a lifelong guarantee. Both the practitioner and the patient must remain vigilant to the possibility of early and late implant failures. Assuming a correctly planned and executed implantation process, early failures commonly result from issues such as improper osseointegration and peri-implantitis. Conversely, late implant failures may be attributed to systemic diseases, infections, radiotherapy, unhealthy habits such as smoking or alcoholism, overloading, traumatic occlusion, parafunctional habits, or psychological factors. It is important to emphasize that dental implants are non-resorbable applications, signifying that once anchored in the cortical bone, they are intended to osseointegrate with the surrounding bone, requiring another surgical procedure for removal.

Among other techniques, coating offers durable and uniform protection, versatility, and cost-effectiveness for large-scale applications, making it suitable for various surfaces. The surface of titanium implants modified by sandblasting, laser processing, and acid etching (SLA surface) is considered the gold standard for preparing implants with high osteointegration potential [3]. The choice of the implant with or without coating depends on specific local factors (such as bone quality) and general factors (such as systemic health) of the patients. In the context of the reviewed article, a nanohydroxyapatite-based coating introduces the desired bioactivity affecting osteoblast differentiation, whereas laser processing or acid etching may not achieve the same goal [3]. The application of coatings on titanium implant surfaces has shown significant potential in enhancing the osseointegration process [5]. This improvement is attributed to the augmented proliferation, differentiation, and mineralization of osteoblasts [6]. Additionally, the accelerated processes of osteogenesis and angiogenesis contribute to a notable reduction in the risk of implant failure [6]. Various coating materials have been explored for their effectiveness in improving the properties of titanium implants. For instance, hydroxyapatite alone has demonstrated positive outcomes, and its combinations with other materials such as silver, chitosan, calcium phosphate, zinc oxide, and collagen have been investigated [1,2]. Application of silver/hydroxyapatite nanoparticle coating has a promising potential to elevate the biocompatibility of dental implants due to the absence of cytotoxic effects, as well as to reduce inflammatory responses after implantation. The combination of nanohydroxyapatite with chitosan revealed a promotion of osteoblast adhesion and differentiation, associated with the proliferation of mesenchymal stem cells. By calcium phosphate coating, a modification of surface topography and subsequent enhancement of osteoblast proliferation can be obtained. Zinc oxide nanoparticles coating manifest a potent antibacterial effect against Escherichia coli and Enterococcus faecalis. Collagen incorporation into the hydroxyapatite coating positively influences cell spreading.

**Figure 1:**
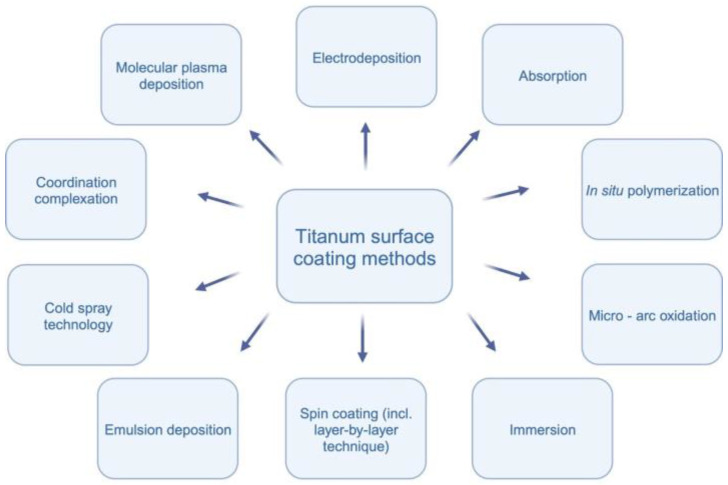
Titanium surface coating methods.

The incorporation of these materials aims to optimize the surface characteristics of titanium implants, providing better outcomes in terms of patient comfort, shorter healing periods, and predictable treatment results (see Figure 1) It is worth mentioning that as an alternative to hydroxyapatite, fluorapatite can be used as coating for titanium [7]. Due to differences in structure and properties, using fluorapatite can be a subject of a separate systematic review [8].

**Figure 2:**
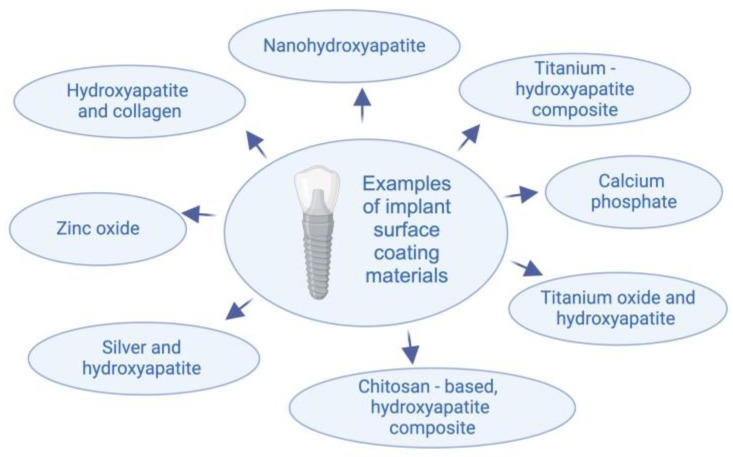
Materials used for implant surface coating.

The application of hydroxyapatite in nanoparticle form demonstrates promising potential for enhancing cell proliferation and improving the osseointegration process on titanium surfaces [9,10,11]. Various techniques for coating titanium surfaces exist, including molecular plasma deposition, electrodeposition, micro-arc oxidation, in situ polymerization, immersion, emulsion deposition, cold spray technology, and spin coating [2,9,10,11,12,13,14,15,16]. Molecular plasma deposition allows precise control over coating thickness, while electrodeposition is cost-effective with good thickness control [12]. Micro-arc oxidation results in a ceramic-like coating, and in situ polymerization enables the incorporation of specific functionalities [16]. Immersion is a simple and cost-effective method, and emulsion deposition provides good coverage with the incorporation of multiple components [9]. Cold spray technology produces dense coatings without high-temperature exposure, and spin coating achieves even coatings through centrifugal force [2]. Each method has unique advantages and challenges, and the choice depends on the desired coating characteristics. Figure 2 visually represents the methodologies employed in the reviewed studies.

Osteoblasts, as primary contributors to the osseointegration process, are integral for orchestrating new bone formation and remodeling [5,6]. Their pivotal role extends to fostering the crucial aspect of achieving robust primary and secondary stability in dental implants, which is paramount for successful healing and treatment outcomes [1,17,18,19,20,21,22,23]. The timeframe between the second and fourth weeks post-implant placement is particularly critical, given that this period is characterized by predominant bone remodeling in cortical bone, while mineralization in cancellous bone is not yet fully established [3]. The strategic functionalization of titanium implant surfaces holds tremendous potential, as it can effectively expedite the proliferation and differentiation of osteoblasts, thereby significantly accelerating the overall osseointegration process [10]. This not only enhances the prospects for achieving accelerated and robust implant stability but also serves as a promising strategy to mitigate the risk of early implant failure [9,10].

In this investigation, our primary aim was to assess the impact of nanohydroxyapatite as a coating material on implant surface functionalization and its subsequent effects on implant properties. Through a rigorous article selection process, adhering to predefined eligibility criteria, we systematically reviewed and synthesized data to identify optimal strategies for modifying implant surfaces. It is important to note that, as of now, a comprehensive systematic review addressing this specific topic is lacking in the existing literature. The completion of such a review not only contributes essential insights but also serves as a potential stimulus for researchers to undertake additional studies. This, in turn, holds the promise of delivering substantial advancements for both implantologists/surgeons and patients in the foreseeable future.

## 2. Materials and Methods

### 2.1. Focused Question

This systematic review adhered to the PICO framework with the following components.

PICO question: In the context of titanium implants (population), does the application of a nanohydroxyapatite layer to its surface (investigated condition) influence the osseointegration process and impact osteoblasts (outcome), as compared to conventional implants without surface functionalization (comparison condition)?

### 2.2. Protocol

**Figure 3:**
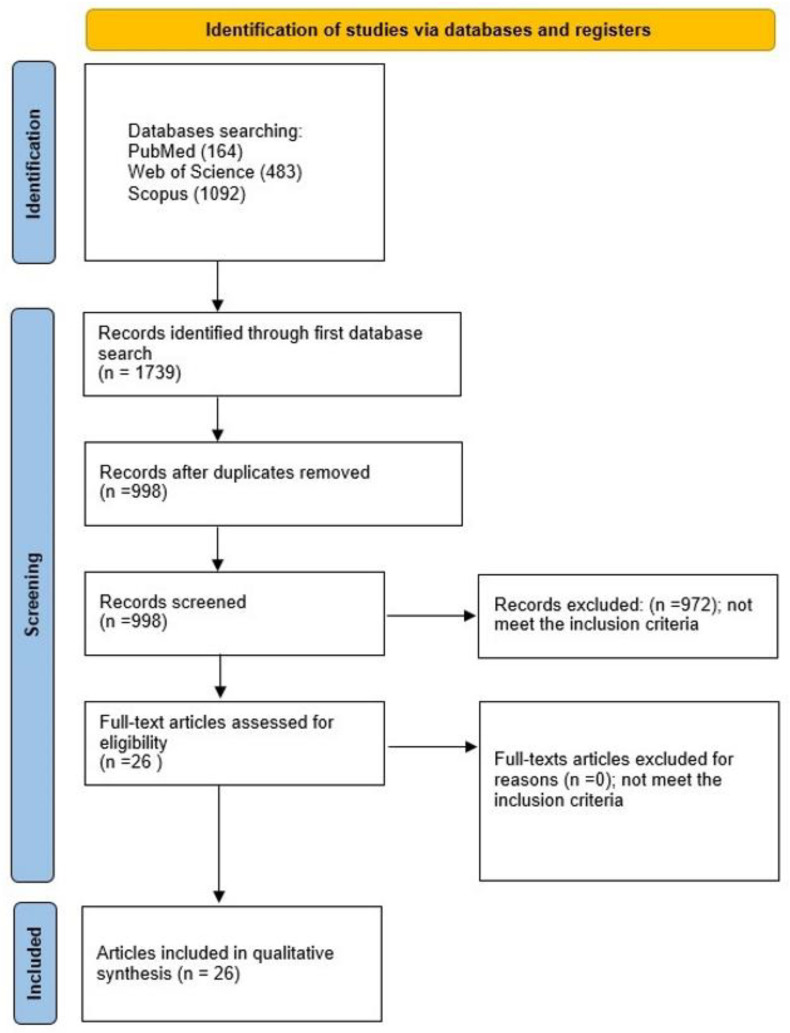
The PRISMA 2020 flow diagram.

The selection process for articles in the systematic review was carefully outlined following the PRISMA flow diagram (see Figure 3).

### 2.3. Eligibility Criteria

All studies included in the systematic review had to meet specific criteria, including: the investigation of nanohydroxyapatite-coated titanium, examination of the impact of nanohydroxyapatite-covered titanium on osteoblasts, inclusion of both in vitro and in vivo studies, involvement of animal and human specimens, and publication in the English language. The reviewers collectively established exclusion criteria, which included studies in non-English languages, clinical reports, opinions, editorial papers, review articles, and studies lacking a full-text version.

### 2.4. Information Sources, Search Strategy, and Study Selection

In November 2023, the authors conducted a search in electronic databases, including Scopus, PubMed, and Web of Science. The authors restricted the results in PubMed and Web of Science to titles and abstracts. In the Scopus database, the search results were limited to keywords, titles, and abstracts. The search criteria were carefully formulated using the keywords ((titanium) AND ((osteoblasts) and hydroxyapatite)). The searches strictly adhered to the established eligibility criteria. After screening abstracts and subsequently reviewing complete full-text versions of articles, only studies related to the utilization of nanohydroxyapatite were included.

### 2.5. Data Collection and Data Items

Six reviewers (K.H., W.Z., P.J.P, A.P., J.K., W.D.) carefully selected articles that met the pre-established criteria. Then, the aforementioned authors collected essential data and organized it in a standardized Excel file.

### 2.6. Assessing Risk of Bias in Individual Studies

During the initial stage of study selection, the authors independently reviewed the titles and abstracts of each study to minimize potential reviewer bias. The level of agreement among reviewers was determined using Cohen’s κ test. Any differences of opinion on the inclusion or exclusion of a study were resolved through discussion between the authors. Risk of bias was assessed based on the quality assessment. 

### 2.7. Quality Assessment

Two independent evaluators (W.Z., J.M.) systematically appraised the procedural quality of each study within the article. The assessment criteria were meticulously designed to focus on critical information pertaining to the nanhydroxyapatite coating on titanium implants. In evaluating study design, implementation, and analysis, rigorous criteria were applied, including a stipulated minimum group size of 10 subjects, the inclusion of a control group, thorough sample size calculation, a precise description of the procedural technique and comprehensive manufacturer’s data, clarification of osteoblast incubation time, and specification of the osteoblasts line used. The studies underwent scoring on a calibrated scale ranging from 0 to 6 points, where a higher score denoted superior study quality. The evaluation of risk of bias followed a meticulous scoring system: 0–2 points indicating a high risk, 3–4 points denoting a moderate risk, and 5–6 points indicating a low risk. Any disparities in scoring were meticulously addressed through thorough discussion until a consensus was achieved.

## 3. Results

### 3.1. Study Selection

A comprehensive evaluation initiated with a total of 1739 studies, from which 741 duplicates were systematically removed. Scrutiny of titles and abstracts resulted in the exclusion of 972 articles that did not align with the specified subjects or objectives. Further refinement during the full-text examination confirmed the inclusion of all papers identified during the initial abstract screening. The final selection for qualitative synthesis comprised 26 studies [1,2,5,6,9,10,11,12,13,14,15,16,17,18,19,20,21,22,24,25,26,27,28,29,30,31], all written in English, and focused on the in vitro assessment of the impact of nanohydroxyapatite-coated titanium on osteoblast activity.

### 3.2. General Characteristics of the Included Studies

The primary objective of this comprehensive systematic review was to meticulously evaluate the implications of implant surface functionalization on the intricate process of osseointegration and its nuanced impact on osteoblast behaviour. The prevalent consensus derived from a substantial body of literature [5,6,11,12,13,14,15,16,17,18,19,20,21,22,23,24,25,26,28,29,30,31] consistently underscores the pivotal role played by implant surface functionalization in augmenting the osseointegration phenomenon. It is imperative to highlight, however, that a singular study conducted by MacBard et al. [2] posited that the application of nanocrystalline hydroxyapatite coating on diverse titanium surfaces might confer limited advantages in cultivating an osteogenic microenvironment. Several investigations [5,6,24,25,26,27,28,29] have directed their focus predominantly towards surface topography and porosity, foregrounding these aspects over the intrinsic properties of coatings. Fernandes et al.’s [5] study distinctly delineated how surface topography significantly modulates osteoblast metabolic activities. Insights gleaned from the investigations by Pourmollaabbassi et al. [6] and Man et al. [29] unveiled that distinct pore sizes and shapes on titanium implant surfaces elicit varied effects on functionalization. Notably, higher porosity and larger pore sizes within HA/TiO_2_ coatings were observed to be conducive to the migration and proliferation of osteoblasts [6]. Furthermore, the shape of the pores emerged as a critical factor; for instance, the triangular pore configuration exhibited a marked enhancement in osteoblast mineralization [29].

A significant corpus of the scientific literature [1,11,12,13,14,16,19,20,21,25,26,30] is dedicated to the meticulous exploration of titanium surface coating with hydroxyapatite in nanoparticulate form and its consequential impact on material properties. The prevailing consensus derived from these studies consistently substantiates that the application of nanohydroxyapatite coating markedly enhances osteogenic properties. In several investigations [9,10,15,16,17,18,20,22,25,27,31], the incorporation of supplementary materials into the hydroxyapatite coating framework has been explored. Chien et al. [15] conducted a study on the dopamine-assisted deposition of hydroxyapatite coating on a titanium substrate, resulting in improved osteoconductivity. Similarly, Kreller et al. [18] treated a titanium surface with a biomimetic calcium phosphate coating, leading to modified surface topography and enhanced osteoblast proliferation. Zhu and colleagues (2010) presented empirical evidence demonstrating that the inclusion of collagen in the hydroxyapatite coating has a positive effect on cell spreading. Two noteworthy studies by Chen et al. [25] and Ma et al. [31] underscored the synergistic osteogenic effects achieved through the combination of nanohydroxyapatite and chitosan. Specifically, Chen et al. [25] scrutinized a structure composed of chitosan-catechol, gelatine, and hydroxyapatite nanofibers, unveiling a promotion of osteoblast adhesion and differentiation, coupled with the proliferation of mesenchymal stem cells.

Ma et al. [31] contribute additional empirical support, affirming the constructive influence exerted by the nanohydroxyapatite/chitosan composite coating on the facilitation of the osseointegration process. According to Qiaoxia et al. [9], the hydroxyapatite coating has heightened antioxidant properties that are critical for reducing reactive oxygen species at the bone–implant interface. The hydroxyapatite/tannic coating, which is characterized by its antioxidant attributes, plays a pivotal role in ameliorating oxidative stress at the bone–implant interface. In two supplementary investigations, Salaie et al. [10] and Fathy Abo-Elmahasen et al. [27], researchers delve into the application of silver/hydroxyapatite nanoparticle coating. Salaie et al. [10] specifically validate the absence of cytotoxic effects, underscoring the potential of silver/hydroxyapatite coating to elevate the biocompatibility of dental implants. Simultaneously, Fathy Abo-Elmahasen et al. [27] unveil a pronounced reduction in bacterial colonies, shedding light on antibacterial properties and proposing the potential of nanocoating as a strategic approach to mitigate inflammatory responses around dental implants (see Table 1).

### 3.3. Main Study Outcomes

Twenty-five studies [5,6,9,10,11,12,13,14,15,16,17,18,19,20,21,22,23,24,25,26,27,28,29,30,31] collectively provide robust support for the hypothesis that nanohydroxyapatite coating significantly improves the chemo-mechanical properties of titanium implant surfaces. This augmentation is intricately linked to increased levels of adhesion, cell proliferation, and osteoblast differentiation, thereby accelerating the complex process of osteogenesis. However, a single study by MacBard et al. [2] departs from this prevailing consensus and suggests that nanohydroxyapatite coating on titanium surfaces may have no discernible effect on osteogenic properties. Within the corpus of these studies, nine [6,12,13,15,18,20,21,24,25] specifically reported the promotion of osteoblast proliferation, while five [1,11,13,17,31] underscored the heightened differentiation of osteoblasts. Six studies [14,15,25,26,28,31] demonstrated an increase in osteoblast adhesion. Notable findings from individual studies include a study [9] confirming that hydroxyapatite coating promotes antioxidant properties and another [27] claiming that hydroxyapatite/silver coating has antimicrobial activity. In addition, three studies [16,19,25] concluded that hydroxyapatite stimulates not only osteogenesis but also angiogenesis (see Table 2).

### 3.4. Quality Assessment

Assessing the risk of bias is essential for ensuring transparency in the synthesis of evidence and the communication of results and findings. The evaluation of study quality, which involves identifying and addressing design flaws, serves as a crucial method for determining the reliability of the studies under consideration. In this scientific endeavor, the authors employed a rigorous approach to assess the quality of the research papers, employing carefully chosen criteria measured on a scale of 0–1, as outlined in Table 3. These criteria encompassed key factors such as group size (at least 10 subjects), presence of a control group, sample size calculation, detailed description of procedure, inclusion of manufacturer’s data, specification of the type of osteoblast line, osteoblast time incubation, and a comprehensive summary of the aforementioned points, forming the basis for calculating the risk of bias. Each criterion present in the assessed scientific work was denoted as ‘1’ in Table 3, while its absence was marked as ‘0’. All the studies were considered to have a moderate risk of bias, with a score of 3/6 [1,2,18,21,25,26,27] and 4/6 [5,6,9,10,11,12,13,14,15,16,17,19,20,22,23,24,28,29,30,31]. A “High risk” rating indicates significant bias that may invalidate the results, on the other hand, “low risk” is considered as a limited amount of bias that could influence the final quality of the research (see Table 3).

## 4. Discussion

As individuals undergo the aging process, the inevitable occurrence of permanent tooth loss becomes apparent. The etiological factors contributing to this phenomenon include periodontal diseases, traumatic incidents such as falls or sports injuries, improper root canal treatments, the absence of permanent tooth buds, and congenital or developmental abnormalities [32,33]. In addressing the restoration of missing teeth, dental implants, as elucidated in reference [16], assume a pivotal role. Conventional dental implants employed for such purposes are fabricated from titanium and its alloys [34]. The strategic utilization of titanium and its derivatives is predicated on their capacity to ensure biocompatibility, corrosion resistance, commendable physical properties, and an effective interaction with body cells, facilitating their adhesion to the implant surface [35,36]. Dentistry, as an ever-evolving scientific discipline, steadfastly pursues the advancement of dental materials and treatment outcomes [37]. Global research endeavours in dental materials, inclusive of implants, are underway to augment their intrinsic characteristics. The present study is poised to scrutinize the nuanced impact of implant surface functionalization on the intricate process of osseointegration. A comprehensive review of the pertinent literature within our investigation consistently underscores the proposition that the deliberate functionalization of the implant surface exerts a direct and substantive influence on the osseointegration process.

Many studies indicated that sandblasting titanium with nanohydroxyapatite alters the surface structure, affecting pre-osteoblasts and directing osteoblast metabolism [5,38,39]. This process creates a roughened surface with increased surface area, which can enhance the osseointegration of the implant. In a separate experiment, titanium-coated HAp nanotubes were produced as an implant coating. The experimental surface exhibited increased osteoblast adhesion and a strong antioxidant effect, with significant potential for ROS removal at the bone–implant interface [9]. After implantation, the surrounding area may experience oxidative stress and bacterial infections, which can lead to apoptosis and eventual bone resorption and implant failure. Free reactive oxygen species are crucial factors contributing to implant loss, owing to the fact that they favour bone resorption. By synergistic antioxidant action of hydroxyapatite and antimicrobial activity of materials like silver or zinc oxide, it is possible to reduce the risk of peri-implantitis development and, in consequence, implant loss [40]. The antioxidant properties of hydroxyapatite were also found by the authors who additionally enriched this material with catechin [41]. It seems that such a modification may have a promising effect, improve the effectiveness of treatment, and prevent the occurrence of peri-implantitis. In one of the experiments, a nanocomposite scaffold was created using Polyurethane sponge, with 50% of its weight being HAp and TiO_2_. The scaffold was used for architectural modeling. The study demonstrated that the P3HB-coated polyurethane sponge possesses mechanical strength and an architecture that is appropriate for osteoblast seeding and growth [9].

To enhance biocompatibility, cell proliferation, and osseointegration, it may be crucial to create suitable architectural structures [6]. Porous surfaces provide more surface area for bone cells to attach and proliferate, which is essential for osseointegration [4]. This is because bone cells can more easily spread out and interact with the implant surface when there are more pores. Other researchers also confirm that pore size influences osteogenesis [42]. Some studies have demonstrated that surface topography significantly affects osteoblast metabolism [43,44]. Moreover, the shape of the pores is also important [29,42,45,46]. The study revealed that implants featuring larger pores, higher interconnectivity, and interconnected macropores demonstrated superior osseointegration when compared to implants with smaller pores, lower interconnectivity, and non-interconnected macropores [47]. Researchers have demonstrated that implants featuring irregular and interconnected pore shapes generally facilitate superior bone formation and osseointegration when compared to implants with smooth or closed-end pores [48].

Some researchers indicate the possibility of incorporating biologically active compounds such as dopamine or calcium phosphate into coatings. This provides titanium implants the biomimetic properties [15,18,49,50,51]. Dopamine forms a self-assembling polydopamine (PDA) layer on titanium surfaces. This layer provides more surface area for bone cells to attach, enhancing their ability to spread and proliferate. PDA carries a negative charge, which aids in cell adhesion and migration. Moreover, it is like the extracellular matrix (ECM), which is the natural scaffold of bone tissue [52,53]. CaP coatings provide an osteoconductive environment for cell attachment, differentiation, and proliferation. CaP coatings facilitate ion exchange between the implant and surrounding bone tissue, promoting the osteointegration process. The release of Ca^2+^ and PO_4_^3-^ ions from CaP coatings stimulates osteoblast differentiation [54,55].

Analysis of the data contained in the publications showed that multidirectional surface functionalization of titanium alloys is highly desirable in contemporary regenerative medicine. Nanohydroxyapatite coatings are totally biocompatible implant elements that aid in improving osseointegration processes. They do not cause adverse reactions or inflammation, creating a favourable environment for bone cells to adhere, grow, and differentiate. Due to the diversity of the research methodology used, the possibility of comparing different functionalization methods is limited. The main limitation of this systematic review is that the samples studied were not kept under the same standardized conditions, because the selected studies were based on both in vitro and in vivo examinations of samples. Establishing standardized protocols for coating preparation, implant fabrication, and evaluation methods would enable more rigorous comparisons of different functionalization approaches. Functionalization of implant surfaces is an interesting research direction and certainly requires further research, including evaluation in clinical conditions.

## 5. Conclusions

Many of the studies assessed in this scientific endeavour affirm that the functionalization of titanium implant surfaces using nanohydroxyapatite enhances osteogenic abilities and expedites the osseointegration process. It is noteworthy that not only does the nanohydroxyapatite coating in isolation impact the overall osseointegration process, but its modifications, such as combining it with materials like collagen, calcium phosphate, or chitosan, can also exert influence. The examined scientific work reveals that modified hydroxyapatite coating additionally augments cell line proliferation, osteogenic activity, or cell spreading, thereby accelerating the entire implantation process. Hence, considering additional modifications to the implant surface becomes imperative, as it contributes to establishing a conducive environment within the tissues upon the modified implant’s contact with the bone. This systematic review, based on the selected studies, unequivocally asserts that hydroxyapatite, whether in its pristine form or modified, especially in the form of nanoparticles, possesses the ability to promote osteoblast adhesion and proliferation. Furthermore, it facilitates their mineralization and ultimately enhances both osteogenesis and angiogenesis processes. The anticipation is that the application of nanomaterials in dentistry, particularly in surgery, will continue to burgeon. The trajectory of medicine stands to gain substantially from the ongoing research on nanomaterials in dental surgery.

## Figures and Tables

**Table 1:** Presents the general characteristics, including the aim of the study, methods and materials employed, results obtained, and conclusions drawn for each research.

Study	Aim of the Study	Material and Methods	Results	Conclusions
Fernandes et al. [5]	Creation of a biointerface, which can control Src-dependent osteoblast metabolism as a pre-requisite to ECM remodeling.	Three titanium discs were investigated, differing in surface properties identified as Machined (Mc; control), Dual Acid-Etched (DAE), and acid-etched nano-HAp-blasted (nHAp). A mouse pre-osteoblastic cell type, was utilized for this study.	Increased MMP activities in response to both DAE and nHAp. Significant increase in Src gene expression and in Integrin, FAK, and Src expression.	Molecular features that are associated with the pre-osteoblast interaction with distinct surface textures can direct Src-dependent osteoblast metabolism as a requirement for extracellular matrix remodeling.
Pourmollaabbassi et al. [6]	Generation of bioartificial bone tissues to overcome issues related to bone loss. Donor site morbidity and size limitations.	HAp powder was derived from bovine bone through thermal analysis at 900 °C. Subsequently, porous HAp with a 50% weight percentage was created using the polyurethane sponge replication method. The cellular scaffolds were divided into four groups for comparative analysis of their behaviour, specifically focusing on osteoblasts. Viability and attachment on the scaffold surfaces were assessed through Methylthiazole tetrazolium (MTT) and Trypan blue analyses as part of the phase studies.	The MTT analysis results of four scaffold groups revealed that Titanium oxide (TiO_2_) did not impact cell growth alone, and HAp was the primary factor for cell growth and osteoblast adhesion on the scaffold. Coating with poly-3-hydroxybutyrate helped retain the factors and position the osteoblasts in the pore.	The correlation between HAp and TiO_2_ is likely to enhance osteoblast adhesion and promote cell growth on the scaffold surface.
Qiaoxia] et al. [9]	Investigation of both the total antioxidant activity and osteoblast behaviour.	Ti^0^ foils served as substrates for growing TiO_2_ nanotube arrays film. The annealed Ti-TiO_2_ samples underwent immersion in a mixed aqueous solution of TA and CaCl_2_, denoted as the TA-CaCl_2_ solution. The morphologies of all coatings were observed using scanning electron microscopy. To assess the viability of mouse pre-osteoblasts on the surfaces of different samples, staining with Calcein-AM and EthD-1 was performed. For proliferation and adhesion experiments, cells (2 × 10^4^/mL) were co-cultured with sterilized samples in 24-well plates. After culturing for 1-, 4- and 7-day(s), cell proliferation was investigated using a Cell Counting Kit-8 (CCK-8) assay.	An antioxidant known as TA enhanced the antioxidative activity of Ti^0^ implants.	The composite coating exhibits antioxidant activity, with significant potential for scavenging ROS at the bone–implant interface. HAp/TA composite coating has superior cytocompatibility in terms of proliferation spreading and adhesion compared to the pure Ti^4+^ substrates.
Salaie et al. [10]	Improvement of the biocompatibility of titanium dental implants coated with Ag^0^ NPs and with HAp applied to their surface.	Titanium discs (Ti6Al4V) are suitably covered by: -Ag^0^ NPs, -Ag^0^ NPs plus nHAp, -Ag^0^ NPs plus mHAp. The coatings were examined for stability and tested for biocompatibility with primary human osteoblasts over a seven-day period.	With the addition of HAp, osteoblasts adhered well and maintained a normal morphology. Lactate dehydrogenase (LDH) leakage was negligible, while alkaline phosphatase (ALP) activity was observed.	The biocompatibility of implants coated with Ag^+^ nHAp was higher than that of those coated with Ag^+^ mHAp or Ag^0^ NPs alone. This indicates that the former may offer clinical advantages.
Cavalcanti et al. [11]	Presentation of valuable insights on implant surface evaluation and demonstration of potential effectiveness of nanostructured material surfaces in, focal cell adhesion, promoting cell viability and bone mineralization in vitro.	Mesenchymal stem cells were utilized for comparative analyses of two different implant topographies, focusing on their functional interaction with pre-osteoblasts directly seeded onto the implants. In vitro assay analysis was used to analyze the functionality of nanostructured implant surfaces. The machined surface of the titanium implant served as a control. It was compared with a surface implant activated by nanoparticle HAp.	Cell culture on the nano-group surface resulted in greater cell adhesion and viability of cultured osteoblasts than in the control group. Scanning electron microscope (SEM) images showed a stable interaction as evidenced by the development of focal cell adhesion.	Nano group was an excellent scaffold for bone–implant integration, together with positive mineralization assays.
Balasundaram et al. [12]	The aim of this in vitro investigation was to produce nHAp and apply it onto titanium (Ti^0^) using molecular plasma deposition (MPD).	nHAp was synthesized via a wet chemical process followed by hydrothermal treatment. Subsequently, nHAp coatings were processed at 500 °C, and micro-sized HAp was processed at 900 °C. SEM, atomic force microscopy, and X-ray diffraction were employed to characterize the coatings before and after sintering.	The results show that the post-MPD heat treatment up to 500 °C successfully restored the structural and topographical integrity of the nHAp. The nHAp-coated substrates supported a greater number of adherent cells compared to both the mHAp-coated and uncoated substrates.	MPD is an innovative method that can be utilized to deposit nHAp on anodized Ti^0^, resulting in higher osteoblast counts relative to untreated Ti^0^ and mHAp-coated Ti^4+^ substrates. MPD is inexpensive, fast, and effective.
MacBard et al. [2]	In vitro comparison of human osteoblasts proliferation on additive manufactured (AM) trabecular-like titanium implant surfaces and traditionally machined base material with titanium plasma spray (TPS)-coated surfaces.	TPS-coated groups: Ti6AL4V ELI machined into discs with diameter of 15 mm and 1.25 mm thick with a 0.75 mm thick TPS-coating on their surfaces. AM groups: discs with diameter of 15 mm and 2.0 mm thick were designed with a solid base (1.25 mm thick and a porous surface layer (0.75 mm thick) and printed by using Ti6Al4V ELI particles through electron beam melting technology. In groups that have HAp coating, a ~20 nm thick nanocrystalline HAp layer was applied on disc surfaces. Number of proliferated cells on discs was measured on day 2, 7, 14, and 21.	Surface of TPS-coated discs: Mean porosity: 59%. Mean pore size: 141.7. Tissue interface height: 743 μm. Estimated average available surface area: 649.39 mm^2^. Estimated surface contact angle: 105.2°. Surface of AM discs: Mean porosity of 60%. Mean pore size: 290.6 μm. Tissue interface height: 1051 μm. Estimated average available surface area: 1153.67 mm^2^. Estimated surface contact angle: 70.1°. Production of calcium on AM discs was 48% higher than on TPS discs.	Additive manufactured trabecular-like titanium implant surfaces encourage faster cell proliferation and higher calcium production than traditionally machined base material with titanium plasma spray coated surfaces. Moreover, nanocrystalline HAp did not enhance osteoblasts proliferation on titanium surfaces.
Shi et al. [13]	In vitro evaluation of osteoblasts proliferation and differentiation on porous titanium implant surfaces coated with nano HAp.	Plates of 10 mm × 10 mm × 1 mm were polished, sandblasted, washed, treated with HF and HNO_3_ solution, then treated with HCl and H_2_SO_4_ solution. Titanium plates were used as a cathode; platinum plates were used as an anode. Electrolytes were prepared by dissolution of Ca(NO_3_)_2_ (0.6 mmol/L) and NH_4_H_2_PO_4_ (0.36 mmol/L) in distilled water to a Ca/P ratio of 1.67. Pre-osteoblasts cells were seeded at a density of 1 × 105 on titanium plates.	Cell proliferation was significantly higher than that in the control group. HAp-coated titanium enhanced alkaline phosphatase activity. Osteocalcin production released into cell culture medium was higher in cells that were grown on HAp surfaces than in those grown on control samples without Hap.	Deposition of nano HAp coating by the electrochemical process improved osteoblasts proliferation and differentiation.
Zhang et al. [14]	Evaluation of properties of helical rosette nanotubes (HRN) and nanocrystalline hydroxyapatite used as coatings on titanium implant surfaces.	Titanium plates 1 cm × 1 cm × 0.2 cm 250 mg of each type of nano HAp (small, middle, large sizes of HAp grains) was mixed with 5 mL of 70% ethanol and sonicated for 20 min. Then, 5 mL of 0.001 mg/mL HRN-K1 solution was added into all samples. Absorption of HRN-K1 and nano HA on titanium surfaces for 45 min. Group without HAp: 0.001 mg/mL HRN-K1 only was used as a titanium coating. Control group: uncoated titanium plates.	Better adhesion of osteoblasts to nanocrystalline HAp and HRN titanium coatings (especially small and middle grain sizes) when compared with uncoated titanium sample. small grains increased adhesion of osteoblast by 29.3%, middle grains by 36.3%.	Nanocrystalline HAp has been shown to have high affinity with HRN. Nanosized HAp and HRN used as a titanium coating promotes adhesion of osteoblasts.
Chien et al. [15]	Development of the procedure for immobilization of Hap nanoparticles and RGD peptides on titanium to improve the osteoconductivity of orthopedic and dental implants.	3-hydroxytyramine hydrochloride was dissolved in 10 mM of Tris buffer to 2 mg/mL. HAp NPs were suspended in 0.02% polyacrylic acid solution. Equal volume of both solutions was mixed and then immediately added onto titanium discs and then incubated for 20 min. Immobilization of RGD: dopamine/HAp coated titanium discs were incubated with 1 mg/mL of RGD solution in phosphate-buffered saline for 24 h.	After 14 days of culture, small (~150 μm)-cell aggregates were found on titanium disc samples, but not on those coated with dopamine only. On the substrate deposited with dopamine/HAp, large (800 μm)-cell aggregates were formed. Dopamine deposition increased calcium amount from 31.6 nmol to 144.3 nmol. On the dopamine/HAp substrate calcium deposition increased to 517.6 nmol.	Co-deposition of dopamine on hydroxyapatite significantly enhances osteoblast mineralization, proliferation and adhesion. RGD peptides immobilized to dopamine/HAp-coated titanium promote osteoblasts adhesion and osteogenic differentiation.
Wang et al. [16]	Investigation of HAp coating of titanium by micro-arc oxidation (MAO).	Titanium surface was modified by MAO, and the sample was named Ti-M. Later, the sample was treated with SHT and placed in a Teflon reactor and then autoclaved at 250 degrees for 1, 4, and 8 h and named as Ti– M–H1, Ti–M–H4, and Ti–M–H8, respectively.	After 0.5 h of incubation the number of cells adhesive on Ti–M– H was significantly increased than that on Ti–M. After longer incubation for 1 h and 4 h, only Ti–M–H1 had a higher number of cells, while Ti–M, Ti–M–H4, and Ti–M–H8 had no significant differences.	MAO- and SHT-treated titanium promote osteogenesis and angiogenesis. It stimulates macrophages to regulate an immune response.
Bezerra et al. [17]	Evaluation of the behaviour of pre-osteoblast on nano HA-blasted titanium surface by examination of intracellular signal pathways.	Three different titanium surfaces were prepared: -machined (control group) -dual acid-etched (DEA) -acid-etched nHAp-blasted (nHAp) Mouse pre-osteoblastic cells were cultured and seeded at the density of 5 × 104 cells/disc on different titanium surfaces.	Ras-Raf-Mek- p42/44 mapk signaling was examined and it was shown to be involved in response to titanium surfaces. Modified surfaces (DAE and nHAp) enhanced an increase in Raf (protein involvement with other signaling pathway). All 3 titanium surfaces stimulated ALP activity in osteoblasts cultured on them.	Different titanium surfaces enhanced crucial intracellular signaling pathways that are responsible for cell proliferation and adhesion. Nanosized HAp-blasted titanium surface promotes pre-osteoblast proliferation by activating crucial signaling pathways.
Kreller et al. [18]	In vitro evaluation of modifying titanium surface with calcium phosphate coating.	Biomimetic CaP coatings were generated by immersion in a modified BCP coating solution at 37 °C for 14 days. Chemically pre-treated titanium plates were soaked in a 1 M of CaCl_2_ solution at 37 °C for 24 h and then immersed in BCP coating solution. Substrates were transferred to BCPx1.5 for 14 days. BCP coating solutions were exchanged to new ones every second day. Control group: chemically untreated titanium surfaces without immersion in BCP coating solution.	Altered surface topographies were obtained due to titanium surface functionalization.	Functionalization of titanium surface with calcium phosphate coatings promotes its osteoconductive properties.
Bai et al. [19]	To describe a microporous TiO_2_ coating decorated with HAp nanoparticles that is generated by micro-arc oxidation of pure titanium and followed annealing	The specimens of a pure titanium were subjected to a micro-arc oxidation fabricating micro/nano-structured surfaces. The process was followed by annealing at different temperatures (250 °C, 450 °C, or 650 °C). Materials were evaluated in terms of osteogenic activity of osteoblasts, angiogenic activity of endothelial cells, macrophage response.	A coating produced with micro-arc oxidation with an annealing temperature of 650 °C exhibits numerous favourable physicochemical properties, such as hybrid micro-nano morphology, superhydrophilicity, and highly crystalline HAp nanoparticles. It supports proliferation and differentiation of osteoblasts and endothelial cells and also inhibits the inflammatory response of macrophages.	The coating annealed at 650 °C exhibited favourable physicochemical properties that synergistically regulated osteoimmunomodulation, osteo/angio-genesis and cross communications amongst immunoregulation, osteogenesis and angiogenesis to significantly enhance osseointegration.
Nakazawa et al. [20]	To assess the physicochemical properties of machined or micro-grooved titanium surfaces which have been coated with titanium-doped HAp nanoparticles and to investigate the subsequent impact on the function of osteoblasts cultured on these surfaces.	Titanium discs with commercially available surface topographies, such as machined or sandblasted, large-grit and acid-etched surfaces, were coated with titanium-doped hydroxyapatite. The discs with original or modified surfaces were evaluated for topography, wettability, and chemical composition. Rat femoral osteoblasts were cultured on the discs and evaluated for proliferation and differentiation.	Titanium hydroxyapatite coatings changed from hydrophobicity to hydrophilicity on both machined and acid-etched surfaces. There was no change in inherent surface topographies on both types of specimens. At day 4, osteoblastic proliferative activity was increased by a Ti^0^-HAp coating.	Titanium-doped hydroxyapatite coating enhances extracellular matrix formation on smooth and microrough titanium surfaces by increasing osteoblastic proliferative activity without compromising differentiation through hydrophilic and chemical functionalization.
Koirala et al. [21]	To synthesize and characterize biomimetic nanosized HAp, using a double emulsion technique combined with a chemical gradient across lipid bilayer for surface modification of a titanium implant.	The synthesized hydroxyapatite was evaluated by: dynamic light scattering, X-ray diffraction, transmission electron microscopy, Fourier-transform infrared spectroscopy, biocompatibility, in vitro proliferation efficacy using Normal Human Osteoblasts.	Nanosized HAp exhibited a diameter of approximately 200 nm with high aqueous stability. The synthesized hydroxyapatite nanoparticles exhibited excellent biocompatibility and provided the cellular proliferative environment. The synthesized material exhibited hierarchical nanostructures inside, filled with nanocrystallites of hydroxyapatite and were highly homogeneous.	Synthesized phospholipid bilayer-coated with hydroxyapatite nanosized construct provides a suitable environment for cell adhesion, particularly important for bone implants. The long-term sustainability of implants would be effectively supported by the incorporation of nanosized hydroxyapatite on the titanium substrate.
Zhu et al. [22]	To characterize synthesized nanocrystalline HAp and evaluate in vitro studies on nano HAp, nano HAp/collagen, and titanium surfaces.	In vitro studies on nanosized HAp, nano-HAp/collagen, and titanium surfaces were performed. Specimens were subjected to: transmission electron microscope evaluation, contact angle assessment, surface roughness evaluation, cell attachment and spreading on The SaOS-2 human osteoblast-like cell line.	Nanosized HAp/collagen was coated homogeneously on pure titanium or porous anodic oxides and exhibited higher wettability. Nano-HAp reduced cell attachment and spreading, and the combination of nano-HAp and collagen showed improved cell spreading of osteoblasts compared to nano-HAp.	To elucidate the reactions at the interface between surfaces and bone, cell responses to nano HAp, nano HAp/collagen, pure and porous titanium surfaces are considered crucial.
Wu et al. [1]	To assess osteogenesis on nano-hydroxyapatite coated TiO_2_ nanotubes on Ti-19Zr-10Nb-1Fe alloy in in vitro tests.	Samples of nano Hap-coated TiO_2_ nanotubes on Ti-19Zr-10Nb-1Fe alloy were characterized by SEM, FT-IR, XRD and assessed in terms of: - Mouse pre-osteoblasts proliferation and adhesion - Alkaline phosphatase and Osteocalcin content assay	TiO2 nanotubes with 87 ± 21 nm were successfully formed on Ti-19Zr-10Nb-1Fe alloy, and nanosized HAp coating was synthesized. The cells’ growth on the surface modified with hydroxyapatite was higher (30% or more) than the unmodified Ti-19Zr-10Nb-1Fe alloy in 7 days, as well as the expression of Alkaline phosphatase and Osteocalcin.	The results suggest that the introduction of nano-HAp improves the differentiation of osteoblasts and the production of local factors, as well as indicating the potential for improved osseointegration of implants.
Vilardell et al. [24]	To evaluate three different surface treatments on a Ti6Al4V alloy for possible application in cementless joint prosthesis in in vitro tests.	Cold Spray technology was used for deposition in all the evaluated methods of surface modifications: (i) an as-sprayed highly rough titanium, followed by the deposition of a thin hydroxyapatite layer with (ii) microcrystalline or (iii) nanocrystalline structure. Primary human osteoblasts, extracted from knee were used to assess cell viability by MTS and LIVE/DEAD assays. Alkaline phosphatase (ALP) was used for cell differentiation test and Phalloidin staining for cell morphology and quantification. All tests were performed at 1, 7, and 14 days of cell culture.	The titanium and HAp surfaces showed different cell morphologies. A higher cell viability was observed on titanium coating. From 7 days of culture, cells on HAp showed good attachment to surfaces and greatly increased proliferation, mostly on nanocrystals, achieving similar cell viability levels to titanium coatings. ALP levels were significantly higher on titanium.	The best cell function results were obtained on titanium coatings. Microcrystalline hydroxyapatite showed the worst cell parameters. Nevertheless, the results suggest that nanocrystalline hydroxyapatite layers could provide promising results for faster cell proliferation once cells have adhered to the surface.
Chen et al. [25]	Investigation of the interaction of osteogenesis and angiogenesis during bone healing in in vitro and in vivo tests using surface-modified titanium substrates with a multi-layered structure composed of chitosan–catechol, gelatine and hydroxyapatite nanofibers as a platform.	The adhesion, morphology, and migration of adipose-derived mesenchymal stem cells (Ad-MSCs) and human umbilical vein endothelial cells (HUVECs) grown on different titanium substrates were evaluated using combined techniques of Transwell co-culture, wound healing assay, enzyme-linked immunosorbent assay (ELISA), quantitative real-time polymerase chain reaction (qRT-PCR), Western blotting and immunohistochemical staining.	Multi-layered titanium substrates directly regulated cellular functions of Ad MSCs and angiogenic HUVEC, mediating intercellular communication through paracrine effects in vitro. The in vivo results showed that the altered microenvironment induced by surface-modified Ti implants promoted the adhesion, recruitment, and proliferation of MSCs and facilitated coupled osteogenesis and angiogenesis in bone healing.	It was proved that multi-layer film-coated titanium substrates positively mediated cellular biological function in vitro and improved bone healing in vivo.
Sato et al. [26]	To improve orthopedic implant performance, the objective of this in vitro study was to synthesize nanocrystalline hydroxyapatite (HA) powders to coat titanium.	HAp was synthesized through a wet chemical process. The precipitated powders were either sintered at 1100 °C for 1 h to produce UltraCap HAp (or microcrystalline size HAp) or were treated hydrothermally at 200 °C for 20 h to produce nanocrystalline HAp. The original HAp particles were characterized using X-ray diffraction (XRD), inductively coupled plasma–atomic emission spectroscopy (ICP–AES), BET, a laser particle size analyser and scanning electron microscopy (SEM). These powders were then deposited onto titanium by a novel room-temperature process, called IonTiteTM.	Results showed increased osteoblast adhesion on the nanocrystalline HAp IonTiteTM coatings compared to traditionally used plasma-sprayed HAp coatings. Results also demonstrated greater amounts of calcium deposition by osteoblasts cultured on Y-doped nanocrystalline HAp coatings compared to either UltraCap IonTiteTM coatings or plasma-sprayed HAp coatings.	These results encourage further studies on nanocrystalline IonTiteTM HAp coatings on titanium for improved orthopaedic applications.
Fathy Abo-Elmahasen et al. [27]	Evaluation of microbiological activities of the deposited nanomaterials (silver/hydroxyapatite nanoparticles (Ag^0^/HAp NPs) and zinc oxide nanoparticles (ZnO NPs)) in terms of microbial inhibition.	Ag/HAp NPs and ZnO NPs were built up onto the surface of titanium OMSs by electrochemical deposition. This electrochemical deposition was performed on 50 orthodontic mini screws. In addition, the microbiological activities of the deposited nanomaterials were explored in vitro in terms of microbial inhibition. Furthermore, the cytotoxicity and cytocompatibility were tested on four types of cells, namely, fibroblasts, osteocytes, osteoblasts, and oral epithelial cells.	ZnO NPs coated OMS had the highest antimicrobial activity than Ag^0^/HAp coated OMS against Gram-positive, Gram-negative, and fungal strains. After incubation, the decrease in the number of bacterial colonies was significant with ZnO and Ag^0^/HAp nanoparticles. ZnO NPs-coated OMSs showed a better cytocompatibility with oral epithelium, bone cells, and fibroblasts compared to Ag^0^/HAp NPs.	Suggested nanocoating is a promising strategy to overcome the development of an inflammatory zone around the fixed OMSs.
Sato et al. [28]	The objective of this in vitro study was to produce nanophase (i.e., materials with grain size less than 100 nm) HAp/titania coatings on titanium.	Nanocrystalline HAp powders were synthesized through wet chemistry and hydrothermal treatments at 200 °C. Nanocrystalline titania powders obtained commercially were mixed with the nanocrystalline HAp powders at various weight ratios. The mixed powders were then deposited on titanium utilizing a room-temperature coating process called IonTiteTM.	The number of osteoblasts adherent on the nanotitania/HAp composite were significantly greater compared with single-phase nanotitania coatings, currently used plasma-sprayed HAp coatings, and uncoated titanium.	These findings suggest that nanosized titania/HAp coatings on titanium should be further studied for improved orthopedic applications.
Man et al. [29]	Investigation of the potential of 3D-printed titanium scaffolds coated with hydroxyapatite to promote the therapeutic efficacy of osteoblast derived Extracellular Vesicles (EVs).	Ti6Al4V titanium scaffolds with different pore sizes (500 and 1000 μm) and shapes (square and triangle) were fabricated by selective laser melting. A bone-mimetic nanosized needle hydroxyapatite (nnHAp) coating was then applied. EVs were procured from scaffold-cultured osteoblasts over 2 weeks and vesicle concentration was determined using the CD63 ELISA. Osteogenic differentiation of human bone marrow stromal cells (hBMSCs) following treatment with primed EVs was evaluated by assessing alkaline phosphatase activity, collagen production and calcium deposition.	Triangle pore scaffolds significantly increased osteoblast mineralization (1.5-fold) when compared to square architectures. Osteoblast-derived EVs isolated from triangular pore scaffolds significantly increased hBMSCs mineralization when compared to EVs acquired from square pore scaffolds (1.7-fold) and 2D culture (2.2-fold) (*p* ≤ 0.001).	These findings demonstrate the potential of harnessing bone-mimetic culture platforms to enhance the production of pro-regenerative EVs as an acellular tool for bone repair.
Yang et al. [30]	Fabrication of a biomimetic lamellar structure on Ti6Al7Nb discs with HAp nanofibers as the intercalated materials and with the Gel and Chi as polyelectrolytes via spin-assisted Lbl assembly technique.	A hierarchical structure with osteoinduction potential was fabricated on titanium alloy (Ti6Al7Nb) substrates via a spin-assisted layer-by-layer assembly technique, with HAp nanofibers as the intercalated materials and gelatine and chitosan as the polycation and polyanion, respectively. The as-synthesized hydroxyapatite nanofibers were characterized using scanning electron microscopy (SEM), transmission electron microscopy, Fourier transform infrared spectroscopy and X-ray diffraction.	Osteoblasts cultured on the hierarchical structure deposited Ti^0^ alloy substrates displayed significantly higher cell viability (*p* < 0.01) and better adhesion, a higher production level of alkaline phosphatase, mineralization, genes expressions of osteocalcin and osteopontin (*p* < 0.01 or *p* < 0.05) compared to those of native Ti6Al7Nb substrates after culture for 4, 7 or 14 days.	The lamellar structure was beneficial for the biological functions of osteoblasts, establishing the basis for osseointegration of a titanium alloy implant.
Ma et al. [31]	Chitosan (CS)-based HAp composites have emerged as a novel strategy for promoting bone regeneration.	Here, nanophase HAp/CS composite-coated porous titanium implants (nCT) were fabricated, and their biological behaviour under diabetic conditions was investigated.	Rat osteoblasts were cultured on bare titanium implants (Ti^0^) and nCT, and subjected to normal serum (NS), diabetic serum (DS), DS + NAC (a potent ROS inhibitor), and DS + cytochalasin D (an actin polymerization inhibitor).	The study illustrated that the reactivation of the FAK-BMP-2/Smad pathway played a crucial role in enhancing osteoblast adhesion and differentiation when utilizing a nano-HAp/CS composite coating.

**Table 2:** Detailed characteristics of included studies.

Authors	Titanium Surface Used in Study	Characteristics of Nanohydroxyapatite and Layer Deposition Technique	Sample Sterilization	Utilized Cell Line	Type of Study	Biological Activity
Fernandes et al. [5]	Machined titanium discs (control), dual acid-etched disc, acid-etched and nanoHAp-blasted discs.	Pro-mimic HAp nano-method (10 nm particles)	Gamma ray sterilization	MC3T3-E1, mouse pre-osteoblastic cells	In vitro	Surface topography affects Src-dependent osteoblast metabolism
Pourmollaabbassi et al. [6]	Nanocomposite scaffold of hydroxyapatite/titanium coated with poly hydroxybutyrate	Hydroxyapatite powder was prepared from bovine bone through thermal technique. Subsequently, a porous HAp material was produced using the polyurethane sponge replication method and coated with poly hydroxybutyrate.	The sample was treated with ethanol under ultraviolet radiation.	The human osteoblast cell lines, Saos-II	In vitro	Higher porosity and large pore sizes of HAp/TiO_2_ scaffold coated with P3HB are preferable for migration and proliferation of osteoblasts.
Qiaoxia et al. [9]	Titanium foils coated by TiO_2_ by electrochemical anodization.	Hydroxyapatite/tannic acid composite, immersion	Performed (no additional data)	The MC3T3-E1 cells	In vitro	HAp/TA coating exhibits antioxidant properties important in eliminating reactive oxygen species in bone/implant interfaces.
Salaie et al. [10]	Polished medical grade titanium alloy (Ti6Al4V) electroplated with Ag NP and HAp coating.	Sintering of the HAp dispersion on the titanium surface.	Gamma irradiation	Human osteoblast cells (HOb)	In vitro	No cytotoxicity in 7 days of cellular culture.
Cavalcanti et al. [11]	Smooth titanium disc and acid-etched with titanium disc with deposited HAp layer.	Pro-mimic nanosized HAp method.	Gamma radiation sterilization	MC3T3-E1 (ATCC 7594) murine osteoblastic cells	In vitro	Enhanced osteoblastic differentiation after exposure to HAp.
Balasundaram et al. [12]	Anodized Ti^0^ foil with and without nano HAp coating.	Molecular plasma deposition of nano HA on anodized Ti^0^.	250 nm UV light exposure	CRL-11372 human osteoblasts	In vitro	Nanosized HAp-coated samples increased proliferation of osteoblasts in comparison with untreated samples and with micron-sized HA coating.
MacBard et al. [2]	Machined Ti6AL4V, TPS coated and with layer of nano HAp.	Dip, spin, and heat treatment technique.	Rinsing in ethanol	hFOB 1.19 fetal osteoblasts-like cells	In vitro	Nanocrystalline HAp coating on different Ti^0^ surfaces provides almost no advantage in generating an osteogenic environment.
Shi et al. [12]	Polished and acid-etched titanium plates covered with HAp coating	Electrolytic deposition of HAp coating.	No data	Murine preosteoblastic cell line (MC3T-E1)	In vitro	HAp coating enhanced both proliferation and differentiation of osteoblasts.
Zhang et al. [14]	Titanium coated with HRN and HAp.	Coating by means of adsorption.	No data	CRL-11372 human fetal osteoblasts	In vitro	HAp/HRN co-coating enhanced osteoblast adhesion
Chien et al. [15]	Ti-6A1-4V titanium alloy coated with HAp/dopamine	Dopamine-assisted deposition of HAp.	Sterilized in 70% ethanol	MG63 osteoblast-like cells	In vitro	HAp coating via polydopamine increased osteoconductivity of the Ti^4+^ substrate. Possibility of incorporation of the RGD peptide.
Wang et al. [16]	Ti^0^ foil with coating for in vitro evaluation and Ti^0^ rods with coating for in vivo evaluation	Micro-arc oxidation and steam-hydrothermal treatment to deposit HAp.	No data	MC3T-E1 preosteoblasts.	In vitro and in vivo	Coating induces osteo- and angiogenesis as well as macrophages’ polarization.
Bezerra et al. [17]	Titanium discs, dual acid-etched Ti^0^ discs and dual etched Ti^0^ discs with HAp coating.	Pro-mimic HAp nano-method.	Gamma irradiation.	MC3T3-E1 mouse preosteoblasts.	In vitro	Upregulation of RUNX2 and ALP biomarkers and promotion of osteoblast differentiation by nHA coating.
Kreller et al. [18]	Ti6Al4V plates coated with biomimetic CaP (HAp).	Immersion BCP technique.	No data	Primary human osteoblasts extracted from the femoral heads.	In vitro	Biomimetic coatings increased cell proliferation and change in concentration of VEGF and IL-6, IL-8, and CICP.
Bai et al. [19]	Ti^0^ sheets and Ti^0^ rods with HAp coating.	HAp deposition through micro-arc oxidation and thermal annealing.	No data	MC3T3-E1 preosteoblasts.	In vitro and in vivo	HAp coating annealed at 650 °C synergistically regulated osteoimmunomodulation, osteo- and angiogenesis to enhance osseointegration.
Nakazawa et al. [20]	Ti^0^ discs coated with Ti^0^/HAp particles.	Spin coating of sol-gel solution of Ti/HAp particles	No data	Rat bone marrow stromal cells extracted from femurs	In vitro	Ti/HAp coating enhanced proliferation and ECM mineralization.
Koirala et al. [21]	HAp-coated Ti^0^ foil	Drop casting and drying nano HA layer deposition	No data	NHOst cells	In vitro	HAp coating provides proliferative environment for the NHOst cells.
Zhu et al. [22]	Oxidized Ti^0^ plates with HAp and HAp/collagen coating.	Wet chemical synthesis of HAp. Deposition of HAp and HAp/collagen coating by immersion.	Ethylene oxide sterilization (42 °C, 12 h)	Human osteoblast-like SaOS-2 cells	In vitro	Addition of collagen to HAp coating enhanced cell spreading.
Wu et al. [1]	TZNF alloy—Ti-19Zr-10Nb-1Fe with HAp coating.	Microsphere-like nanohydroxyapatite crystals formed by double anodic oxidation	UV sterilization	Mouse pre-osteoblasts, MC3T3-E1	In vitro	Promotion of osteoblast differentiation.
Vilardell et al. [24]	Titanium 6Al4V alloy, coated using a cold spray technology	Microcrystalline or nanocrystalline hydroxyapatite layer	Sterilization in ethanol	Primary human osteoblast cells (HOBs) extracted from knee trabecular bone	In vitro	Surface area affects cell attachment. Higher proliferation increased levels of ALP.
Chen et al. [25]	Multi-layered titanium structure fabricated with layer-by-layer technique using a spin coater.	Structure composed of chitosan–catechol, gelatine, and hydroxyapatite nanofibers.	Thermal sterilization at 121 °C for 20 min.	Adipose-derived mesenchymal stem cells and human umbilical vein endothelial cells.	In vitro and in vivo	Promotion of adhesion, recruitment, and proliferation of mesenchymal stem cells and facilitated coupled osteogenesis and angiogenesis in bone healing.
Sato et al. [26]	Titanium (no additional data) with HAp coating	Nanocrystalline hydroxyapatite synthesized hydrothermally and sintered microcrystalline size hydroxyapatite	No data	Human osteoblast-like cells, CRL- 11372.	In vitro	Increased osteoblast adhesion on the nanocrystalline hydroxyapatite.
Fathy Abo-Elmahasen et al. [27]	Titanium mini-screws, OMS system for skeletal anchoring coated with HAp.	Silver hydroxyapatite nanosized particle coated by electrodeposition.	No data	Normal primary cells isolated from a human jaw: fibroblasts, osteocytes, osteoblasts and oral epithelial cells.	In vitro	Decreased number of bacterial colonies.
Sato et al. [28]	Titanium (no additional data).	Nanocrystalline hydroxyapatite/titania synthesized by wet method and hydrothermal processes. Coated in room-temperature in a IonTite process.	Steam autoclave sterilization.	Human osteoblast-like cells, CRL- 11372.	In vitro	Promotion of osteoblast adhesion.
Man et al. [29]	3D-printed (selective laser melting) titanium scaffolds with different pore sizes and shapes.	A bone-mimetic nano-needle hydroxyapatite.	UV and autoclave sterilization.	MC3T3 murine pre-osteoblasts and human bone marrow stromal cells.	In vitro	Some of the scaffolds (e.g., triangle pore) significantly increased osteoblast mineralization.
Yang et al. [30]	Titanium 6Al7Nb disks.	Hydroxyapatite nanofibers prepared by hydrothermal synthesis	No data	Osteoblasts isolated from the newborn rat’s cranium	In vitro	Beneficial for the biological functions of osteoblasts.
Ma et al. [31]	Porous Ti6Al4V frameworks.	Nanohydroxyapatite/chitosan composite coating.	No data	Primary rat osteoblasts.	In vitro and in vivo	Promotion of osteoblast adhesion and differentiation and eventual osteointegration was observed.

**Table 3:** Assessing risk of bias, presence (1) or its absence (0).

Authors	Group Size at Least 10 Subjects	Control Group	Sample Size Calculation	Detailed Description of Procedure, Manufacturer’s Data	Type of Osteoblast Line	Osteoblast Time Incubation	Total Points	Risk of Bias
Fernandes et al. [5]	0	1	0	1	1	1	4	Moderate
Pourmollaabbassi et al. [6]	0	1	0	1	1	1	4	Moderate
Qiaoxia et al. [9]	0	1	0	1	1	1	4	Moderate
Salaie et al. [10]	0	1	0	1	1	1	4	Moderate
Cavalcanti et al. [11]	0	1	0	1	1	1	4	Moderate
Balasundaram et al. [12]	0	1	0	1	1	1	4	Moderate
MacBard et al. [2]	0	0	0	1	1	1	3	Moderate
Shi et al. [13]	0	1	0	1	1	1	4	Moderate
Zhang et al. [14]	0	1	0	1	1	1	4	Moderate
Chien et al. [15]	0	1	0	1	1	1	4	Moderate
Wang et al. [16]	0	1	0	1	1	1	4	Moderate
Bezerra et al. [17]	0	1	0	1	1	1	4	Moderate
Kreller et al. [18]	0	0	0	1	1	1	3	Moderate
Bai et al. [19]	0	1	0	1	1	1	4	Moderate
Nakazawa et al. [20]	0	1	0	1	1	1	4	Moderate
Koirala et al. [21]	0	1	0	1	1	0	3	Moderate
Zhu et al. [22]	0	1	0	1	1	1	4	Moderate
Wu et al. [1]	0	0	0	1	1	1	3	Moderate
Vilardell et al. [24]	0	1	0	1	1	1	4	Moderate
Chen et al. [25]	0	1	0	1	0	1	3	Moderate
Sato et al. [26]	0	0	0	1	1	1	3	Moderate
Fathy Abo-Elmahasen et al. [27]	1	0	0	1	1	0	3	Moderate
Sato et al. [28]	0	1	0	1	1	1	4	Moderate
Man et al. [29]	0	1	0	1	1	1	4	Moderate
Yang et al. [30]	0	1	0	1	1	1	4	Moderate
Ma et al. [31]	0	1	0	1	1	1	4	Moderate

## Data Availability

Not applicable.
